# Supplementary material for: Are carbon emissions trading and green financial instruments synergistic? -Comprehensive quantitative research based on content analysis
Source: PLoS One. 2024 Mar 7;19(3):e0298601. doi: 10.1371/journal.pone.0298601 (PMC10919601; doi:10.1371/journal.pone.0298601)
Supplement: S1 Data — (ZIP) [file pone.0298601.s001.zip › Data/地级市Z_F工作报告.zip!/╣π╬≈/║╪╓▌╩╨/└┤╫╘╣1⁄2╓┌║┼í╛┬φ┐╦╩2╛▌═°í┐_2005.docx]

各位代表：

　　现在，我代表市人民政府向大会报告工作，请予审议，并请市政协各位委员和其他列席会议的同志提出意见。

2004年工作回顾

　　2004年，是我市经济社会发展取得显著成效的一年。在市委的正确领导下，在市人大及其常委会和市政协的监督、支持下，市人民政府和全市各族人民一道，高举邓小平理论和“三个代表”重要思想伟大旗帜，全面贯彻落实党的十六大和十六届三中、四中全会精神，以科学发展观为指导，坚定不移地推进“三化两业”，克服了持续干旱、煤电油运紧张、项目建设用地偏紧等困难，较好地完成了市一届人大三次会议确定的各项任务,使全市继续保持了经济快速增长和各项事业全面发展的良好势头。过去的一年，是我市近十年来经济发展最快、经济增长亮点最多、经济运行质量和效益最好、城乡居民收入增幅最大的一年。

　　──经济快速发展，“三突破”目标全面实现。初步统计，全市生产总值132.1亿元，增长12.6%，实现了“九五”以来的最快增长；财政收入8.78亿元，增长24.4%，增幅居全区第一；规模以上工业企业盈亏相抵后实现利润1.8亿元，增长37%。以上三项指标均超额完成了自治区下达的“三突破”目标任务。

　　──三次产业全面增长，经济结构实现质的转变。一、二、三次产业分别增长4.1%、24.4%和10.1%，第二产业占GDP的比重由上年的33.1%提高到37.2%，首次超过第一产业上升为第一位，实现了三次产业结构由“一二三”向“二一三”的历史性转变，改变了我市长期以来以传统农业为主的经济结构格局。

　　──重大项目建设实现新突破，经济发展后劲不断增强。全市新开工规模以上项目699项，比上年增加251项。全社会固定资产投资46.6亿元，增长107.1%，增幅居全区各市前列。特别是对贺州经济社会发展全局具有深远影响的广贺高速公路贺州段奠基，洛湛铁路贺州段、贺江复航工程开工建设，桂梧高速公路贺州段、国道323线莲塘至鹰阳关二级公路建设加快推进，拉开了“南北贯通,五龙出海”大通道建设的序幕，贺州将结束没有铁路、高速公路和港口的历史，交通条件将发生质的变化，区位优势将进一步提升，加快发展的基础将越来越牢固。

　　──招商引资硕果累累，外向型经济加快发展。全市引进内资项目745个，利用外来资金39.6亿元，增长152.5%；引进外资项目18个，实际利用外资2686.8万美元，增长30.4%。外贸出口9387.4万美元，增长4.3%。

　　──就业再就业超过预期目标，城乡人民生活水平稳步提高。全市城镇新增就业1.1万人，城镇登记失业率3.75%，控制在4.5%的预期目标内。城镇居民人均可支配收入8890元,增长10.8%。农民人均纯收入2089元，增长10.3%。全社会消费品零售总额25.6亿元，增长12.2%。人口自然增长率7.3‰，控制在预期目标内。

　　──社会和谐稳定，社会主义物质文明、政治文明和精神文明共同进步。全市呈现出经济繁荣，各项社会事业全面进步，社会稳定，民族团结，广大干部群众聚精会神搞建设、齐心协力谋发展的可喜局面。

　　这些成就，表明我市的经济建设和社会事业已经步入快速发展的关键时期。

　　一年来，市人民政府主要做了以下九个方面的工作：

　　第一，坚持优先发展工业，工业成为经济增长的主导力量。我们一手抓现有优势产业和强优企业的做大做强，一手抓新优势产业的培育，努力发挥工业对经济增长的主要推动作用。完成了桂东电子100万平方米中高压铝箔、灵峰药业GMP认证与年产20万箱金鸡系列中成药等技改项目。全市工业企业技改项目实现新增工业产值17.8亿元，利润5263万元。发挥工业园区集聚效应，积极接纳东部产业转移，加快发展外向型企业和民营企业。旺高工业区、信都工业基地新引进项目20个，投产5个。新引进的广西大锰银鹤公司年产1.2亿只电池生产线、贺州奥奇丽公司年产10万吨洗衣粉、贺州金昌丰稀土公司年产2500吨高纯稀土功能材料等一批工业项目开工建设。深化国有企业改革，完成西湾矿务局的关闭破产以及市第二机械厂等一批国有企业改制工作。加大对工业经济运行的监控和协调管理，最大限度地缓解煤电油运对工业生产的制约，工业经济运行质量和效益明显提高。全市工业增加值38.2亿元，增长18.2%。

　　第二，加快城市建设步伐，城镇化水平有新提高。组织实施“两年一小变”城市建设方案，完成了贺州市城市总体规划、城镇体系规划、江南新区控制性详规、贺州学院新校区详细规划等专项规划，启动了全市工业发展规划、城市风貌规划的编制工作。城市基础设施建设力度加大，城市功能进一步完善。龟石水库供水一期工程、八达西路改造、恒泰花园、市中医医院综合楼等项目竣工，市体育中心、管道燃气供气工程等项目开工建设。开展了灵峰山保护专题调研，召开了专家论证会、各方代表参加的听证会，努力解决灵峰山的保护与开发问题。进一步推行门前“三包”责任制，市容市貌有了较大改观。三个县城的建设和改造也取得新成绩。

　　第三，着力解决“三农”问题，农业和农村经济稳步发展。

　　强化对农业的支持和保护，加大农业结构调整力度，县域经济发展加快， “三农”工作呈现出多年来少有的好形势。优势农产品基地规模扩大，无公害农产品行动计划取得新成绩。畜牧水产业稳步增长，为农民增收发挥了重要作用。农业企业化深入推进，新增规模农业企业50家，新建农民专业合作经济组织20家。开通鲜活农产品运输“绿色通道”，促进农产品流通。林业生态建设进一步加强，速丰林基地稳步发展。以龟石灌区节水改造为标志的农田水利基础设施建设与改造取得新进展，农村生产生活条件进一步改善。积极抓好防汛抗旱工作，努力降低灾害损失。

　　第四，大力发展商贸旅游业，第三产业整体水平稳步提高。完成了全市旅游总体规划、富川秀水状元村旅游资源开发与保护详规、钟山荷塘风景区保护与开发等规划的编制工作。引进桂林旅游股份有限责任公司开工建设了贺州温泉二期改造工程。姑婆山国家森林公园旅游国债项目顺利推进，贺州温泉、玉石林景区二级公路动工建设。旅游宣传促销力度加大，行业管理逐步规范，旅游业服务质量和水平不断提高。全市接待游客243.5万人次，增长33.1 %，其中境外游客8.04万人次，增长94.2%；旅游总收入9.4亿元，增长25.2%。商贸业快速发展，盛隆百货、新时代广场、桂湘粤日用杂货批发市场、粮油农贸市场、汽车农机市场、金旗市场等一批规模大、档次高的商贸设施建成使用或开工建设，促进了商贸经济的活跃繁荣。金融保险业快速健康发展，对地方经济建设的促进作用不断增强。

　　第五，加快推进项目建设，发展环境进一步改善。继续实施百项工程大会战，狠抓项目跟踪服务，集中开展项目征地拆迁工作，努力保障城乡建设、重点项目建设所需资金和用地，扩大基础设施、优势产业和社会事业投资规模，进一步优化投资结构，发挥了投资对经济增长的拉动作用。基本建设投资增长85.1%，更新改造投资增长75.8%，房地产投资增长39.9%。事关贺州发展大局的“南北贯通,五龙出海”通道项目以及金牛坪水电站、上程水电站等一批基础设施和产业项目相继开工建设，增强了经济发展后劲。

　　第六，加大招商引资和发展民营经济工作力度，引进利用市内外资金实现了新突破。坚持把招商引资和发展民营经济作为充分吸纳市内外资金，加快贺州发展的有效途径，抓住中国—东盟自由贸易区加快构建和泛珠三角区域经济合作加强的机遇，积极推进区域合作，不断创新招商引资手段，组织开展洽谈会招商、专业队招商、挂职招商、以商招商等多形式招商活动，成功举办了桂东电子产品订货暨经贸项目洽谈会、贺州市首届农产品展示暨农业项目洽谈会和广州、珠海民营企业家座谈会等重大招商活动，招商引资取得了重大突破。与此同时，认真落实鼓励、支持和引导民营经济发展的政策措施，放手发展民营经济。全市私营企业户数增长31.6%，从业人数增长10.4%，注册资本增长22.9%。

　　第七，深入实施六项“民心工程”，困难群众生活得到改善。年内新建五保村417个，已累计兴建五保村649个，安置五保老人9635人。年内安排1518名大中专毕业生就业。城镇下岗失业人员再就业3662人。全年培训农村富余劳动力10.4万人次，新增劳务输出2.9万人。少数民族地区脱贫致富工程和扶贫开发工作取得新成果，又有1.25万人解决了温饱问题。开展创建“平安贺州”活动，全面推进社会治安综合治理，深入开展严打整治斗争，社会治安状况继续好转，全市治安案件和刑事案件发案率分别下降13.48%和5.76%。认真做好信访和人民调解工作，妥善处置群体性事件，积极预防和化解社会矛盾纠纷，社会稳定工程取得明显成效。加强安全生产专项整治，各类事故起数、死亡人数分别下降16.2%和9.5%。社会保障工作明显加强，社会保险覆盖面扩大，“两个确保”进一步巩固，全市企业离退休人员养老金按时足额发放。我们还高度重视解决土地征用、房屋拆迁、企业改制、农民工工资、教育收费、困难企业军转干部经济待遇、招招聘教师、医疗服务中存在的问题，切实维护人民群众的利益。

　　第八，统筹兼顾协调推进，社会各项事业蓬勃发展。组织实施第二轮科技创新计划46项，科技对经济的支撑作用增强。“两基”教育成果得到巩固提高，中小学校布局调整和危房改造工作取得新进展。普通高中办学规模继续扩大，办学质量提高。申办贺州学院工作稳步推进，贺州学院新校区建设进展顺利。高度重视人才工作，加强人才队伍建设。文化事业繁荣发展，艺术精品创作再创佳绩。深入开展“扫黄打非”工作，出版物市场和文化市场进一步净化，新闻出版和广播电视事业有新的发展。市、县疾控中心和传染病区（科）国债项目加快建设，公共卫生服务体系初步建立，重大疾病预防控制取得新进展。全民健身活动广泛开展，八步区沙田镇荣获全国亿万农民健身活动先进乡镇称号。以奖励扶助为主的农村计划生育利益导向机制逐步建立和完善，人口与计划生育工作在巩固中提高，我市连续两年获得全区人口与计划生育目标管理党政线考核一等奖，昭平县荣获全国人口与计划生育优质服务先进县称号，八步区和昭平县、钟山县均被评为全区人口与计划生育目标管理达标先进单位，富川县巩固了人口与计划生育一类县成果。强化矿产资源开发秩序的治理整顿，严厉打击非法采选矿行为，努力建立长效管理机制，矿业秩序进一步好转。加强城市环境综合治理，开展“贺江清新行动”，贺江水质有较大改善，生态环境质量提高。加强市场监管，开展食品安全专项整治。深入推进“桂东文明绿洲”建设，城乡精神文明水平进一步提高。国防动员、民兵和预备役建设继续加强，双拥工作迈出新步伐，荣获自治区双拥模范城称号。依法加强民族、宗教事务管理，人事、机构编制、技术监督、供销合作、民政、审计、物价、统计、气象、人防、档案、修志、外事、侨务、妇女、儿童和残疾人等工作取得新成绩。

　　第九，加强政府自身建设，行政能力进一步提高。各级政府和各部门以开展“创建学习创新型领导班子”、“学习广东，加快发展，我为贺州作贡献”等活动为契机，围绕“廉洁、勤政、务实、高效”的目标，加快政府职能转变，认真贯彻实施行政许可法，深化行政审批制度改革，深入开展机关效能建设，增强了机关的创新力和执行力，办事效率和服务水平进一步提高。加强民主法制建设，不断完善科学民主决策机制。自觉接受人大依法监督和政协民主监督，认真办理人大代表建议和政协提案。进一步加强廉政建设，开展行风评议活动，政风建设得到加强。

　　各位代表，在宏观环境趋紧、区域竞争加剧以及土地、资金、能源和重要原材料制约较严重的情况下，取得以上成绩，确实来之不易。这是自治区党委、自治区人民政府和市委科学决策、正确领导的结果，是市人大有效监督、大力支持，市政协积极参政议政、民主监督的结果，是全市人民团结一致、顽强拼搏的结果。在此，我代表市人民政府，向全市各族人民，向驻贺人民解放军和武警部队官兵、公安干警，向各民主党派、工商联、人民团体以及所有关心、支持贺州建设和发展的各界人士，表示衷心的感谢并致以崇高的敬意!

　　回顾过去一年的工作，有几条经验体会值得我们认真总结和继续坚持：第一，必须坚持以发展为第一要务，抢抓国内外经济持续发展的难得机遇，着力解决经济社会发展中的突出矛盾和问题，千方百计加快发展步伐，努力保持较快的增长速度。第二，必须坚持以项目为载体，抓投入促发展，注重抓好新上产业项目和技改项目，优化经济结构，发展有市场、有效益的优势产业和特色产业，加快转变经济增长方式，提高经济增长质量。第三，必须坚持“借助外力加快发展”和“主动融入粤港澳经济圈”的理念，高度重视抓好招商引资，抓住中国─东盟自由贸易区加快构建、泛珠三角区域经济合作加强和西部大开发等机遇，优化发展环境，创新招商引资机制，不断提高招商引资成效，为经济社会发展注入强大动力。第四，必须以科学发展观统领经济社会发展全局，统筹推进经济社会协调发展，坚定不移地以人为本，竭尽全力为民办实事、办好事，切实维护好、发展好、实现好人民群众的根本利益，全力维护社会稳定。

　　在充分肯定成绩的同时，我们也清醒地看到，我市经济社会发展中还存在不少困难和问题，主要是：经济结构性矛盾仍然比较突出，工业主导作用仍不够强，工业企业效益有待进一步提高；农业基础设施还不完善，抗灾减灾能力不强，农民收入水平还不高；煤电油运和资金、土地等供需矛盾比较突出，对经济发展的制约较大；经济总量偏小，财政收支矛盾依然突出；城镇就业再就业压力加大，安全生产形势依然严峻；政府职能转变力度有待于进一步加大，经济发展的软环境还有待进一步改善等等。我们将高度重视这些问题，在今后的工作中采取措施，努力加以解决。

2005年主要任务

　　2005年是实施“十五”计划的最后一年，也是我市加快推进“三化两业”，乘势而上，加快发展的关键一年。政府工作的总体要求是：以邓小平理论和“三个代表”重要思想为指导，深入贯彻党的十六大、十六届三中、四中全会和全区经济工作会议精神，全面落实科学发展观，抓住当前有利时机，继续以财政增长、农民增收为基本目标，以项目为载体，以发展非公有制经济和外向型经济为突破口，打通道、降门槛、强队伍、引项目、建基地，按照“五业并举，工业优先，夯实基础，做大总量”的要求，牢固树立“工业立市”思想，加快推进工业化、城镇化、农业企业化，巩固提高商贸旅游业，保持经济快速发展的良好态势，努力提高城乡居民生活水平，大力发展各项社会事业，切实加强精神文明和民主法制建设，努力构建和谐社会，推动经济社会全面协调可持续发展，实现社会主义物质文明、政治文明和精神文明共同进步。

　　全市经济社会发展的主要预期目标建议为：生产总值增长10%以上；财政收入增长12%以上；全社会固定资产投资增长20%以上；全社会消费品零售总额增长12%；外贸出口额增长7%；城镇居民可支配收入增长6%；农民人均纯收入增长6%；城镇新增就业增长5%；人口自然增长率控制在7.8‰以内。

　　要实现上述目标，必须着力抓好以下十个方面的工作：

　　一、牢固树立“工业立市”思想，强化工业主导地位

　　以项目为中心，培育壮大强优企业和优势产业。一是加大技改项目实施力度，促进产业优化升级，做大做强电力、林产、制药、电子、冶炼、建材、农副产品加工等优势产业和强优企业。重点抓好桂东电子年产500万平方米中高压电子铝箔扩建，加快发展上下游产品，延长产业链；加快推进贺达纸业年产10万吨高级文化纸和林浆纸一体化项目、灵峰药业年产50万箱金鸡系列中成药等技改项目，力争全市全年完成技改投资5.38亿元，增长30%。积极创造条件，促进电力、林纸、林化、林板等四大集团的组建和发展，加快培育一批年销售产值10亿元、创税1亿元和年销售产值5亿元、创税5000万元以上的骨干企业。二是全力推进一批新的工业项目开工建设，培育新的优势产业。抓好矿业的规划和管理，整合开发矿产资源，加快建设石材加工、有色金属加工、粉体材料深加工三大矿产品加工基地，培育发展有色金属及稀有金属深加工、石材开采与加工、非金属粉体加工、水泥生产、铁锰综合开发五大支柱矿业和产业集群，力争用3年时间使矿业年产值超过50亿元，成为我市新的支柱产业。三是加快奥奇丽公司年产10万吨高档洗衣粉、大锰银鹤公司年产1.2亿只电池生产线、金昌丰稀土公司年产2500吨高纯稀土功能材料等在建工业项目建设，争取尽快建成投产。四是加强水电能源项目建设，努力解决电力能源瓶颈制约问题。加快上程电站、下福电站、金牛坪电站等一批在建水电站的建设步伐，争取尽快建成发电。加强与广西电力公司的合作，增加外购电量。进一步抓好城乡电网建设与改造，加大电力企业生产挖潜力度，缓解电力供求矛盾。

　　以园区为载体，培育经济增长新亮点。认真研究“一区多基地”的运作模式，把信都、西湾工业基地纳入旺高工业区进行规划建设，盘活用好平桂、西湾矿区等国有闲置土地。加大园区招商引资力度，做好企业入园工作，加快入园项目建设，做大做强园区经济。旺高工业区今年新入园企业15家以上，投产企业10家以上，完成销售产值5亿元以上，创税3000万元以上。

　　以改革为动力，盘活存量资产。全力推进以产权制度为核心的国有企业改革，积极推进股份制和股份合作制，大力发展混合所有制，重点推进梧州松脂厂等企业的改制工作，力争年底前全面完成国有企业改制任务。健全各级国有资产监督管理机构，完善国有资产管理体制和监管方式，对所监管的企业实行严格的经营业绩考核制度。严格执行国家法规和政策，防止国有资产流失，维护出资人和职工的合法权益。

　　二、加快城市建设步伐，着力塑造城市新形象

　　坚持规划先行，完善城乡规划。做好贺州市城市总体规划、土地利用总体规划的完善和报批工作，完成城市风貌规划，做好洛湛铁路贺州火车站站前广场和大道、市区太白湖公园和西约街、新宁街等旧城区控制性详规以及城市供排水、电力、地下管网等专项规划，开展市内城区现状测绘工作。抓紧修编完善桂梧高速公路、广贺高速公路、洛湛铁路贺州段沿线乡镇发展规划，推进小城镇建设，加强农村建设的规划管理。坚持依法按规划进行城镇建设。

　　全面加快交通基础设施建设，提高交通对经济社会发展的支撑力和贡献力。按照“南北贯通,五龙出海”的要求，从今年起实施为期3年、总投资超112亿元的交通基础设施建设大会战。年内开工建设广贺高速公路贺州段和八步至黄姚、富裕至马江二级公路，加快推进洛湛铁路贺州段、桂梧高速公路贺州段、昭平至蒙山黄村二级公路等续建项目建设，争取国道323线莲塘至鹰阳关二级公路和富川至钟山、恭城至富川三级公路贺州段建成通车。积极争取将钟山至湖南道县高速公路列入泛珠三角经济圈高速公路网规划，全面完成农村等级公路改扩建项目前期工作，加快乡村公路建设，提高乡村道路通达能力。启动贺州港信都作业区、昭平作业区建设，按计划整治贺江、桂江航道。加强道路运输站场建设，加快建设市客运中心，完成信都二级客运站项目前期工作，动工建设16个乡镇客运站。

加快市政基础设施建设，完善城市功能。狠抓 “三年一中变” 城市建设方案的实施。争取启动江南新区一期工程路网建设，构筑起江南新区框架。加快江北东路建设，推进光明大道、星光路、爱民路、万兴路等道路的延伸建设。抓好贺州大道、建设路等市区道路的改造。建设八达西路延长线，促进西湾工业基地建设，进一步扩大市区规模。建设贺州商业步行街区，重点抓好新宁街、体育路等街道的改造。加快龟石水库供水二期地下管网工程、贺州学院新校区、管道燃气供气工程等在建项目建设。推崇新的城市房地产开发理念，推广住宅小区、经济适用房的开发，开工建设汇豪国际城、香港商贸城、中华园住宅小区、灵峰花园等一批房地产开发项目，积极培育房地产市场，做大做强房地产业，使之成为经济增长的新亮点。推进生活垃圾无害化处理厂、城市污水综合处理工程、人防工程等项目建设，进一步完善城市基础设施。重视小城镇的规划、建设与管理，抓好各县城区的建设，加快发展信都、西湾、望高、黄姚、朝东等重点镇，促进城乡统筹发展。进一步加大依法征地拆迁工作力度，妥善解决好征地拆迁工作中存在的问题，确保城镇建设项目的用地。

　　推进城市经营，加强城市管理。通过积极争取国家开发银行和外国政府贷款，有偿有期转让基础设施经营权，多渠道筹集城市建设资金。抓好城区国有闲置土地的处置和开发。以参加全区第五届市容市貌“南珠杯”竞赛和创建全区文明县城为契机，大力开展城市容貌、交通秩序、市场秩序、房屋出租等专项整治，完善城市管理长效机制，确保创城夺杯。加强对灵峰山、大钟山等城区自然景观的保护，实施净化、绿化、亮化、美化工程，努力创造洁净、优美、舒适的生活环境。

　　三、加强“三农”工作，加快推进农业企业化

　　推进农业结构调整，发展优势特色农业。在稳定粮食种植面积，确保粮食安全的前提下，加快发展优势经济作物产业群，大力发展畜牧水产业，推进无公害蔬菜、脐橙、梅李、茶叶、马蹄、红瓜子、烟叶、瘦肉型猪、优质肉牛、速生丰产林、松脂林、中药材、食用菌等优势农产品基地建设，促进特色农产品向规模化、标准化、无公害化方向发展，努力打造全国“脐橙之乡”、“梅李之乡”、“茶叶之乡”和全区优质肉牛种源基地。

培育和发展龙头企业，加快推进农业企业化。坚持运用创办工商企业的理念谋划农业发展，落实好各项扶持政策，培育壮大梧州松脂、贺达纸业、三金畜牧水产、西牛牧业、金犊种畜、大成食品、将军峰茶叶公司等一批以农产品加工为重点的骨干农业龙头企业。培育扶持一批农产品出口企业，积极发展农产品出口基地，拓展出口市场。加大农业招商工作力度，积极引进知名农业龙头企业，力争今年新增规模农业企业50家。加强龙头企业与农民的利益联结，加快发展订单农业。加大专业合作经济组织、流通大户、农民经纪人队伍的培育扶持力度，推进农民专业协会试点示范工作，提高农民进入市场的组织化程度。继续完善鲜活农产品运输“绿色通道”建设，促进农产品流通。

　　加快生态农业示范市建设步伐，推进农产品无公害标准化生产。抓好市农产品质量安全检测分中心、动物产品检测中心资质认证工作，提高检测中心和已建立的乡镇流动检测站综合检测能力，争取新建1－2个县级农产品质量安全检测站和一批乡镇级流动检测站。继续实施无公害农产品行动计划，建设无公害标准化生产示范基地38个、面积50万亩，标准化养殖示范场200个。做好名优农产品申报认定工作。加快发展生态富民家园，大力推广“猪+沼+果+灯+鱼”等生态农业模式，全年建设沼气池1.8万座。切实加强动植物疫情监测，建立健全检疫防疫网络，完善防疫应急措施，增强处置紧急疫情的能力，确保我市无重大动植物疫情发生。

　　加强农业综合生产能力建设，增强农业发展后劲。认真贯彻落实《中共中央国务院关于进一步加强农村工作提高农业综合生产能力若干政策的意见》，积极争取国家各项支农资金，努力增加各级财政对农业的投入。抓好农田水利、人畜饮水、农村电网、乡村道路等农村基础设施建设，改善农业发展环境。继续实施好龟石水库灌区续建配套和节水改造工程。加快农业科技创新和农技推广应用体系建设，提高农业科技含量。加快实施沃土工程，切实提高耕地质量，提高农业综合生产能力。

　　深化农村改革，稳定、完善和强化各项支农政策。落实最严格的耕地保护制度，继续推进农村税费改革，农业税税率降至2%。继续落实好种粮直补、农机具补贴、退耕还林以及改善农民工进城就业环境等扶持政策。深化粮食流通体制改革，全面放开粮食收购市场和销售市场。推进林业改革，加快林地使用权的合理、合法流转。依法加强林业资源管理，抓好生态公益林的保护。推进以县级联社为突破口的供销合作社改革。组织实施好乡镇撤并工作。依法稳定农村土地承包经营机制，深化土地征收制度改革。

　　大力发展县域经济，增强县（区）经济实力。继续把发展县域经济作为解决“三农”问题、推动城乡协调发展的重要举措，把着力点放在落实各项扶持政策，增强县域自我发展能力上。以发展民营经济为突破口，加大招商引资工作力度，加快推进农业企业化，大力发展农产品加工业和优势资源型加工业，着力培育能够带动和支撑县域经济发展的优势产业和支柱产业，注重发展乡镇经济，增强县级财政自主增长能力，缓解财政困难。

　　四、巩固提高生态旅游业，繁荣发展商贸服务业

以景区景点建设为重点，全力打造旅游品牌。以建设生态型商贸旅游城市和文明风景旅游区为目标，按照“提升品位、形成合力、打造品牌、快速发展”的总体要求，整合开发旅游资源，抓好重点旅游景区景点配套设施建设，着力打造旅游品牌。重点推进姑婆山国家森林公园二期国债项目、黄姚古镇景区建设项目、钟山碧水岩、荷塘十里画廊、姑婆山旅游综合服务基地以及贺州温泉二期改造工程，加快临贺故城、秀水状元村等新景区景点的保护与开发。加强区域旅游协作，积极融入珠三角、大桂林旅游圈，创新旅游宣传促销方式，包装策划好旅游“黄金线路”，拓展旅游客源市场。继续加强旅游景区景点综合环境建设和旅游饭店星级标准化建设，整顿和规范旅游市场，加强从业人员素质培训，提高旅游服务水平，力争全市接待游客人数、旅游总收入均增长10%以上。

　　以有形市场建设为重点，加快发展商贸服务业。开工建设建材市场、农机市场、城北小商品市场、鼎富装饰广场等一批专业市场，加快桂湘粤日用杂货批发市场、新时代广场等在建项目建设。搞好市区商业网点规划，优化商业网点布局。适应市场需求，加快发展金融保险、信息服务、公共交通等现代服务业，积极发展社区服务、中介服务业，改造提高商贸、宾馆、餐饮、娱乐等传统服务业。积极引进大型贸易流通企业，培育发展一批物流龙头企业，加快发展现代物流业。进一步完善服务业结构，提高服务业层次和水平。继续整顿和规范市场经济秩序，深入开展打击制售假冒伪劣食品、药品、农资等违法犯罪活动。

　　五、深入实施百项工程大会战，努力扩大投资规模

　　完善工作机制，集中力量推进重大项目建设。进一步完善推进重大项目建设的责任机制和激励机制，统筹推进一批重大项目开工建设。全年新开工投资规模500万元以上的105个项目，完成投资31亿元以上。加快在建项目建设进度，全年续建投资规模2000万元以上的46个项目，完成投资48亿元以上。创新重大项目前期工作机制，加大前期工作经费投入，加快推进一批具有区域优势、资源优势的重大项目前期工作。强化项目管理，全面实行项目法人责任制、招投标制、工程监理制和合同管理制，确保工程质量。

　　培育壮大多元投资主体，多渠道筹措建设资金。运用市场机制筹措建设资金，加快引进和培育壮大多元投资主体，形成促进民间投资的激励机制、基础设施建设的良性循环机制和灵活高效的资本运作机制。加大招商引资工作力度，引进更多的国内外资金。积极推动银企合作，促进银行扩大贷款规模。积极争取国债资金、中央预算内资金、财政专项资金和政策性贷款。有计划地把政府投资的基础设施项目向社会转让经营权或股权，将回收的资金进行再投资，增强政府扩大投资能力。

　　用足用活国家土地政策，多方解决项目建设用地问题。抓好土地利用总体规划的调整，进一步做好土地储备工作，做到储备和开发并重，盘活平桂、西湾矿区和国有农林场的国有土地，抓紧对存量土地和闲置厂房、仓库进行全面清理，尽可能多地提供建设用地。加紧已批土地项目的落户工作，决不允许征而不用、征后缓用或征多少用。

　　六、加大招商引资工作力度，全面提高外向型经济发展水平

　　加强和改进招商引资工作，提高招商引资成效。进一步完善招商引资的目标责任、激励机制、考核办法，强化招商专业队伍建设，不断创新招商方式，继续组织开展洽谈会招商、专业队招商、挂职招商、委托招商、以商招商等行之有效的招商活动，注意把招商引资的重点区域由粤港澳台地区逐步向闽苏浙地区和东盟国家扩展，着力抓好浙江的招商引资系列活动，与浙江的市、县政府和商会签订经济合作协议，加强区域合作。加大“百企入桂”工作力度，办好亚细安（东盟）客属第六届恳亲会、石材产品订货暨项目洽谈会、贺州市第二届农产品展示暨农业项目洽谈会等活动，使之成为我市对外开放和招商引资的重要平台。加强招商引资项目的跟踪、协调和服务，提高合同履约率、项目开工率、资金到位率和投产率。对已达成意向和正在洽谈项目，落实专人跟踪，加强督促、指导和服务，争取更多的意向和在谈项目尽快落户。突出抓好已签约项目的落实，协助做好项目报批、招投标、征地拆迁等前期工作，使项目尽早开工建设。引进项目要与保护自然资源相结合，严禁引进高能耗、高污染、低效益的项目。加强投资软环境建设，落实好各项优惠政策，着力解决外来投资者反映的突出问题，营造亲商、富商、安商的创业环境。

积极开拓国际市场，努力扩大外贸出口。充分利用外贸环境改善的有利条件，优化出口商品结构，推进出口市场、外贸经营主体、贸易方式多元化。在巩固传统出口市场的同时，全力开拓东盟市场。加强对重点出口企业的指导和服务，大力抓好松香、锡锭、金属硅、林化产品等传统优势产品出口。用好国家扶持政策，支持企业扩大出口，帮助更多的中小企业、个体私营企业获得进出口经营权，更多地出口马蹄、脐橙等农副产品和陶瓷、大理石、竹木制品等本地产品，培育新的出口增长点。

　　加大扶持、引导力度，加快发展民营经济。认真落实鼓励、支持和引导民营经济发展的政策措施，放宽市场准入，努力创造平等竞争、一视同仁的法治环境、政策环境和市场环境。重点扶持发展科技型、外向型、吸纳就业型、农产品加工型民营企业，鼓励和引导民营企业创立品牌、创新技术、创新体制、规范管理。改善对民营企业的金融服务，增加有效信贷投入，促进中小企业发展。加强行业协会建设，发挥行业协会对民营经济发展的促进作用。维护民营企业的合法权益，依法保护公民合法私有财产。

　　七、做好财税金融工作，增强资金保障能力

　　做好组织收入工作，确保财政增长。大力培植财源，提高财政收入的质量。把项目建设作为财政增收的重要环节来抓，加快项目建设进度,尽可能多地完成投资量。继续深化国有企业产权制度改革，盘活国有资产存量，增加国有资产经营收入。坚持有偿开发原则，挖掘优势资源的增收潜力。进一步完善组织财政收入的激励机制，强化收入目标责任制。规范和强化税收征管，堵塞税收漏洞，确保应收尽收。加强非税收入管理，规范执收行为。同时，利用国家财政政策调整和自治区调整对下财政体制的机会，加强与上级的联系，争取更多政策资金支持，缓解县乡财政困难。

　　优化支出结构，确保收支平衡。按照科学发展观统筹安排财政支出。在保证国家规定的工资津贴正常发放、国家机关正常运转、扶贫、救灾救济、疾病防治等政策性支出的同时，努力增加对“三农”工作、社会保障、文教卫事业发展的投入，保证各项重点支出，严格控制一般性支出。严格执行财政预算，加强财政资金支出管理。抓好部门预算的编制工作，继续深化国库集中收付制度和政府采购管理制度改革。

　　加强银企合作，支持地方经济发展。加强与金融部门的沟通协调，积极向银行推介优势产业、重点项目和强优企业，争取扩大贷款规模。积极向上级争取金融机构升格和扩大信贷规模，提高金融服务水平，发挥金融对地方经济发展的促进作用。

　　八、落实科学发展观，统筹推进各项社会事业发展

　　积极推进科技创新。实施科教兴市战略，抓好第三轮科技创新计划的实施，加快新产品、新技术的引进、开发和应用，加快科技成果的转化。做好昭平县作为全国科技工作试点县以及昭平、钟山县作为全区县域科技创新试点示范县的实施工作，继续抓好科技示范基地和科技示范乡镇、示范村、示范户的建设。积极发展高新技术产业和民营科技企业。

优先发展教育事业。各级政府要用更多的财力和更多的精力抓好教育工作。统筹城乡教育发展，进一步完善农村义务教育管理体制和教育经费投入保障机制，严格实行义务教育“一费制”，巩固提高“普九”水平。加强农村教育，调整农村小学布局，提高农村初中办学水平。实施农村中小学危房二期改造工程，改善办学条件。加快发展高中阶段教育，协调发展普通高中与职业技术学校。大力发展幼儿教育、成人教育。继续做好贺州学院的申办工作，积极创造条件创办贺州高等职业技术学院。抓好教师队伍建设。鼓励和支持社会力量办学，加强民办教育的管理。

　　加强多层次人才队伍建设。认真实施人才强市战略，抓好培养、吸引和使用三个环节，重点加强行政管理人才、企业经营管理人才和专业技术人才三支队伍建设。推进人才小高地建设，加强中高级技能人才培养。推进人事制度改革，落实和促进人才发展的各项政策措施，创建鼓励优秀人才脱颖而出、人尽其才的有效机制和良好环境。筹建贺州人才市场，促进人才合理流动。

　　加快完善公共卫生体系。建立健全突发公共卫生事件应急机制，进一步完善疾病预防控制体系、医疗救治体系和卫生执法监督体系，加快公共卫生国债项目建设，提升疾病预防控制能力。加强农村医疗卫生基础设施和农村卫生队伍建设，搞好富川县新型农村合作医疗制度试点，做好农村贫困人口医疗救助制度实施工作。推进农村妇幼保健和改水、改厕、改变居住环境工作，强化食品、药品安全监管。纠正医药购销和医疗服务中的不正之风。

　　加快其他各项事业发展。繁荣发展文化事业和文化产业，加大基层文化建设力度，加强文化市场建设和管理，推进区域性文化共建活动。大力开展群众性体育活动，提高竞技体育水平，积极发展体育产业。抓好市文化艺术中心、市体育中心等文化体育设施建设。加强广播电视网络建设，实施自然村“村村通”广播电视工程。加快新闻出版业的发展，深入开展“扫黄打非”，积极推进政府系统软件正版化工作。搞好经济普查工作。关心和支持老龄、妇女、儿童、残疾人等事业。继续做好民族、外事、侨务、对台、统计、民政、气象、防震减灾、档案、修志等工作。切实搞好国防教育、国防动员、人民防空工作和民兵、预备役部队建设，做好复员转业军人的安置工作，扎实开展军民双拥共建活动，争创全国双拥模范城。

　　加强人口、资源和生态环境工作。进一步建立和完善以奖励扶助为主的农村计划生育利益导向机制，稳定低生育水平，提高出生人口素质。加强土地利用的管理，深入开展矿产资源管理秩序治理整顿工作，创新矿产资源管理机制。合理开发和节约使用资源，大力发展循环经济，提高资源综合利用率。深入开展“贺江清新行动”，强化环境综合治理和环境保护。

　　九、加强社会主义精神文明和民主法制建设，努力构建和谐社会

　　加强民主法制建设。坚持贯彻依法治国方略，严格执行国家法律。自觉接受人大及其常委会的监督，认真执行人大及其常委会的各项决定和决议，定期向人大报告工作，支持人大代表依法行使职权。加强与人民政协的协商，接受人民政协的民主监督。认真办理好人大代表议案、建议和政协委员提案。广泛听取各民主党派、工商联和无党派人士的意见，发挥工会、共青团、妇联等人民团体的桥梁纽带作用。依法保护妇女儿童、残疾人和未成年人的合法权益。继续推行政务公开、厂务公开和村务公开，强化基层民主监督。精心组织全市村委会、居委会换届选举工作，加强农村基层政权建设和城市社区建设。加强普法工作，提高公民法律素质，增强公务人员的法制观念和依法办事能力。

加强精神文明建设。深入贯彻公民道德建设实施纲要，加强社会公德、职业道德、家庭美德和爱国主义教育，加强未成年人思想道德建设。深入开展以“桂东文明绿洲”为载体、以文明城市、文明乡镇、生态文明村、文明社区、文明行业和文明单位为重点的精神文明创建活动，大力提倡团结互助、见义勇为的良好风尚。搞好科普宣传，弘扬科学精神，倡导文明健康的生活方式。

　　努力改善困难群众生活。继续实施六项“民心工程”。全面完成721个五保村建设任务，进一步完善五保村管理机制。实施好农村贫困残疾人危房改造工程。高度重视就业工作，加大就业岗位开发力度，帮助下岗失业人员再就业。加强农村富余劳动力培训，扩大劳务输出。全年培训农村劳动力10.5万人次，新增劳务输出2.45万人。继续抓好少数民族地区的扶贫开发，全年解决1.2万人农村贫困人口的温饱问题。继续巩固“两个确保”，做好“三条保障线”的衔接工作，规范和完善城市“低保”。完善基本养老保险制度，继续推进医疗保险制度改革，全面实施《工伤保险条例》，扩大社会保险覆盖面，加大社会保险的征缴力度，加强社保基金的监督管理，加快完善社会保障体系。努力帮助困难群众解决好就医、就业、子女就学难等问题。

　　全力维护社会稳定。深入推进“平安贺州”和“三无”社区、村屯创建活动，加强依法治理工作，加快社会治安打防控体系建设，建立健全群防群治网络，严厉打击各种违法犯罪活动。做好信访和人民调解工作，集中处理好信访突出问题及群体性事件，积极预防和化解各种矛盾纠纷。重视处理好国有企业改制等涉及群众利益的问题，做好建筑领域拖欠工程款、农民工工资的清理工作。加强安全生产监督管理，全面落实安全生产责任制和各项政策措施，开展安全生产专项整治，坚决遏制重特大事故发生。

　　十、加强政府行政能力建设，提高管理经济社会的水平

　　全面履行政府职能，努力建设务实高效政府。加快政府职能转变，全面履行经济调节、市场监管、社会管理和公共服务职能。积极推进政府决策的科学化、民主化，制定和出台重要决策前，要深入调查研究，广泛征求意见，认真组织做好“十一五”规划的编制工作，使“十一五”规划的编制过程成为科学民主决策的过程，使我们的决策更加符合贺州的实际，更加符合人民的利益。深化行政审批制度改革，深入开展机关效能建设，健全和完善行政效能考核标准体系，提高行政效能，降低行政成本。

　　全面推进依法行政，努力建设法治政府。深入贯彻实施行政许可法和全面实施依法行政纲要，严格依照法定的权限和程序行使权力、履行职责，依法管理经济社会事务。依法保障人民群众的合法权益，自觉接受人民群众的监督。加强和改进行政执法，切实解决有法不依、执法不公、多头执法等群众反映强烈的问题。加强行政执法监督，加强行政执法人员素质培训，促进严格执法、公正执法和文明执法。

　　加强政风建设，努力建设人民满意的政府。进一步转变工作作风，多深入基层搞好调查研究，为基层和群众解决实际问题。改进会风和文风，会议和文件要比上年减少三分之一以上。继续完善和落实经济目标责任制，强化督查考核，确保完成各项工作任务。加强廉政建设，落实廉政建设责任制，严格执行领导干部经济责任审计和重点资金、重点项目、重点工程审计，健全土地出让、建设项目招投标、资产交易和政府采购制度，从源头上预防腐败。坚决查处违法违纪案件。在政府机关深入开展保持共产党员先进性教育活动和创建学习创新型领导班子活动，建立健全公务员和领导干部学习培训制度，不断加强理论武装、知识更新和能力培养，特别是要重点学习、研究和借鉴广东、浙江等先进地区的发展经验，提高工作创新力、执行力，努力建设一支政治坚定、业务精通、清正廉洁、作风优良的公务员队伍，不断开创政府工作新局面。

　　各位代表，做好今年各项工作，任务艰巨，意义重大。让我们紧密团结在以胡锦涛同志为总书记的党中央周围，高举邓小平理论和“三个代表”重要思想伟大旗帜，进一步解放思想，抢抓机遇，真抓实干，全面完成今年各项工作任务，为实现全市经济社会的跨越式发展，全面建设小康社会而努力奋斗！
